# Interleukin-12 as a biomarker of the beneficial effects of food restriction in mice receiving high fat diet or high carbohydrate diet

**DOI:** 10.1590/1414-431X20187900

**Published:** 2018-11-14

**Authors:** C.B. de Almeida-Souza, M.M. Antunes, G. Godoy, C.R. Schamber, M.A.R.C.P. Silva, R.B. Bazotte

**Affiliations:** Departamento de Farmacologia e Terapêutica, Universidade Estadual de Maringá, Maringá, PR, Brasil

**Keywords:** Pro-inflammatory cytokines, Anti-inflammatory cytokines, Serum cytokines, Caloric restriction, Nutrition

## Abstract

The impact of food restriction (FR) during 56 days on serum levels of cytokines in mice fed a high-fat diet (HFD) or high-carbohydrate diet (HCD) were evaluated. The amount of food was reduced 50% for HFD-FR and HCD-FR groups compared to mice receiving free access to HFD (HFD group) or HCD (HCD group). We quantified the serum levels of basic fibroblast growth factor, granulocyte-macrophage colony-stimulating factor, inducible protein 10, interferon γ, interleukin 1α (IL-1α), IL-1β, IL-2, IL-4, IL-5, IL-6, IL-10, IL-12, IL-13, IL-17, keratinocyte chemoattractant, macrophage inflammatory protein-1α, monocyte chemotactic protein 1, monokine induced by IFN-γ, and tumor necrosis factor α. Only IL-12 levels were lower (P<0.05), for both HFD-FR (HFD-FR *vs* HFD) and HCD-FR (HCD-FR *vs* HCD). Therefore, IL-12 levels could be considered a biological marker of the beneficial effects of FR.

## Introduction

Excessive caloric consumption as a high-fat diet (HFD) or high-carbohydrate diet (HCD) is largely responsible for the epidemics of chronic diseases associated with inflammation. In fact, inflammation has emerged as an important aspect of the pathophysiology of chronic diseases including obesity, metabolic syndrome, and cardiovascular diseases (1).

The relationship between high caloric consumption and inflammation is well established (2,3). Furthermore, the reduction of caloric intake improves immune response and antioxidant activity, reduces lipid accumulation in liver, suppresses pro-inflammatory cytokines, modulates energy balance, and extends lifespan in animal models (4–6).

In spite of the anti-inflammatory effects of food restriction (FR), there is a lack of data demonstrating if the blood level of a specific cytokine could better express the beneficial anti-inflammatory effects of caloric restriction.

To achieve this purpose, the impact of FR in mice receiving HFD or HCD during 56 days on serum levels of basic fibroblast growth factor (FGF-basic), granulocyte-macrophage colony-stimulating factor (GM-CSF), inducible protein 10 (IP-10), interferon γ (IFN-γ), interleukin 1α (IL-1α), IL-1β, IL-2, IL-4, IL-5, IL-6, IL-10, IL-12, IL-13, IL-17, keratinocyte chemoattractant (KC), macrophage inflammatory protein-1α (MIP-1-α), monocyte chemotactic protein 1 (MCP-1), monokine induced by IFN-γ (MIG), and tumor necrosis factor α (TNF-α) were evaluated.

## Material and Methods

### Animals and treatments

Male Swiss mice (*Mus musculus*) weighing approximately 35 g were used in the experiments. They were housed in a room with a controlled temperature of 23°C and an automatically controlled photoperiod (12-h light/12-h dark).

The experimental protocol was approved by the Animal Ethics Committee of University of Maringá (1067160216/CEUA) and was in accordance with international laws on the protection and use of animals.

The mice were randomly divided into four groups (n=5-9). HCD and HFD groups had free access to food while the amount of food was reduced 50% for HCD-FR or HFD-FR for 56 days. During this period, all mice had free access to water. The diets were purchased from Rhoster Company (Brazil). The diet composition was based on purified diets for maintenance of laboratory adult rodents (AIN-93-M) proposed by the American Institute of Nutrition (7).

The ingredient compositions of the HCD and HFD, the details about the fatty acid composition, and chemical composition of the HCD and HFD can be found in our previous publication (3).

After 56 days of food restriction (HCD-FR and HFD-FR groups) or free access to food (HCD and HFD groups), the mice were fasted overnight (15 h) and euthanized by decapitation. Blood was collected, centrifuged at 1235 *g* for 10 min at 4°C to obtain the serum, and stored at -80°C until the measurements of cytokines.

### Measurements of serum cytokines

FGF-basic, GM-CSF, IP-10, IFN-γ, IL-1α, IL-1β, IL-2, IL-4, IL-5, IL-6, IL-10, IL-12, IL-13, IL-17, KC, MIP-1-α, MCP-1, MIG, and TNF-α were evaluated with the mouse cytokine magnetic 20-Plex panel (Novex® by Life Technologies, USA) with the immunoassay Luminex Magpix Platform (Luminex Corporation, USA), as previously described (8).

### Statistical analysis

Results were analyzed by Student's *t*-test to assess differences between HCD *vs* HCD-FR or HFD *vs* HFD-FR and are reported as means±SD. P-values less than 0.05 indicated statistical significance. Graph-Pad Prism (USA) Version 5.0 software was used for the analyses.

## Results and Discussion

The inflammation triggered by obesity involves many components of the classical inflammatory response to pathogens and includes increased blood inflammatory adipokines, recruitment of leukocytes from inflamed tissues, and generation of reparative tissue responses. In addition, there is an overexpression of inflammatory genes associated with obesity and metabolic diseases in adipocytes. These multiple inflammatory mechanisms contribute to the increased pro-inflammatory circulating cytokines (9).

Caloric restriction reduces the production of inflammatory cytokines, systemic inflammation, liver steatosis, and insulin resistance (4). Since adipose tissue releases pro-inflammatory adipokines, low adiposity through caloric restriction could reduce inflammatory responses (10).

In the present study, we investigated the effects of food restriction on serum pro-inflammatory and anti-inflammatory cytokines levels in mice. The initial body weight for all groups was similar (results not showed). The body weight gains (means±SD, n=10) were 19.0 g ± 5.2, 5.5 g ± 3.0, 15.6 g ± 5.4, and 11.1 g ± 3.6, for HCD, HCD-FR, HFD, and HFD-FR, respectively. As one would expect, there was less intense (P<0.05) body weight gain (HCD *vs* HCD-FR or HFD *vs* HFD-FR) in mice submitted to food restriction.

We observed three patterns of response to food restriction (Tables 1 and 2). First, there was absence of statistical differences in the serum concentrations of cytokines (HFD *vs* HFD-FR or HCD *vs* HCD-FR) for FGF-basic, IL-1α, IL-1-β, IL-2, IL-4, IL-5, IL-6, IL-10, IL-13, IL-17, IP-10, MCP-1, MIG, MIP-α, and TNF-α. Second, statistical differences (P<0.05) were found in the serum levels of cytokines GM-CSF, IFN-γ, and KC (HFD *vs* HFD-FR).

**Table 1.** Serum levels of cytokines (pg/mL) in freely fed high-fat diet (HFD) or food restricted high-fat diet (FR-HFD) mice for 56 days.

Cytokines	HFD	FR-HFD	% change
FGF-basic	138.9±6.8	163.0±19.7	17.3
GM-CSF	5.7±0.9	9.4±0.7*	65.4
IFN-γ	9.3±1.1	15.0±2.6*	61.1
IL-1α	5.3±1.5	4.4±0.6	-17.5
IL-1-β	79.0±1.8	75.8±1.9	-4.1
IL-2	14.3±0.3	13.5±0.6	-5.3
IL-4	12.9±2.7	18.1±5.9	40.9
IL-5	8.3±2.8	8.2±3.2	-0.5
IL-6	4.1±0.2	4.8±0.5	16.3
IL-10	19.8±1.9	19.4±2.4	-1.6
IL-12	60.4±3.8	36.5±5.7*	-39.6
IL-13	192.8±2.0	203.5±11.4	5.5
IL-17	1.0±0.2	0.9±0.2	-7.7
IP-10	5.2±0.8	4.3±0.7	-16.4
KC	165.7±19.5	300.7±23.3*	81.4
MCP-1	26.3±2.9	22.5±1.8	-14.7
MIG	20.1±2.1	26.6±2.9	32.3
MIP-α	33.6±2.8	26.9±2.0	-19.9
TNF-α	3.7±0.4	3.0±0.3	-19.0

Data are reported as means±SD, n=5-9. FGF-basic: basic fibroblast growth factor; GM-CSF: granulocyte-macrophage stimulating factor; IFN-γ: interferon gamma; IL: interleukin; IP-10: inducible protein 10; KC: keratinocyte chemoattractant; MCP-1: monocyte chemotactic protein-1; MIG: monokine induced by IFN-γ; MIP-α macrophage inflammatory protein-alfa; TNF-α: tumor necrosis factor alfa. *P<0.05 FR-HFD *vs* HFD (Student’s *t*-test).

**Table 2.** Serum levels of cytokines (pg/mL) in freely fed high-carbohydrate diet (HCD) or food restricted high-carbohydrate diet (FR-HCD) mice for 56 days.

Cytokines	HCD	FR-HCD	% change
FGF-basic	145.1±8.3	140.1±7.2	-3.4
GM-CSF	9.0±0.6	9.7±0.9	7.3
IFN-γ	21.6±3.2	14.9±2.5	-31.3
IL-1α	3.4±0.7	3.1±1.3	-9.3
IL-1-β	81.8±2.6	76.8±2.7	-6.1
IL-2	15.0±0.3	13.8±0.5	-8.1
IL-4	16.7±2.0	14.2±0.1	-15.0
IL-5	14.8±2.3	10.0±3.4	-32.5
IL-6	7.2±1.6	7.2±1.0	-0.4
IL-10	15.2±2.6	18.6±2.3	22.2
IL-12	78.0±4.4	50.0±3.6*	-38.2
IL-13	199.4±2.6	199.8±3.3	0.2
IL-17	1.2±0.3	0.8±0.1	-35.2
IP-10	3.7±0.7	4.6±0.7	24.5
KC	241.2±44.1	254.7±35.2	5.6
MCP-1	23.9±2.0	23.1±1.5	-3.5
MIG	36.0±3.5	30.6±3.7	-15.0
MIP-α	32.6±1.8	31.9±1.9	-1.9
TNF-α	3.2±0.3	3.3±0.4	2.3

Data are reported as means±SD, n=5-9. FGF-basic: basic fibroblast growth factor; GM-CSF: granulocyte-macrophage stimulating factor; IFN-γ: interferon gamma; IL: interleukin; IP-10: inducible protein 10; KC: keratinocyte chemoattractant; MCP-1: monocyte chemotactic protein-1; MIG: monokine induced by IFN-γ; MIP-α macrophage inflammatory protein-alfa; TNF-α: tumor necrosis factor alfa. *P<0.05 FR-HCD *vs* HCD (Student’s *t*-test).

Since caloric restriction prevents chronic inflammation, the increased (P<0.05) serum levels of GM-CSF, IFN-gamma, and KC (HFD *vs* HFD-FR), represent an unexpected result. However, the terms pro-inflammatory and anti-inflammatory cytokines in general oversimplify the highly complex physiopathological process. For example, increased IFN-γ and KC mediated the protective effects of FR in hippocampus (11) and neutrophils (12), respectively. Furthermore, the increased production of GM-CSF by NK cells of C57BL/6 mice submitted to FR is critical to prevent viral infections (13). Also, GM-CSF has a dual role working as a pro-inflammatory or anti-inflammatory cytokine (14). Therefore, the higher (P<0.05) serum levels of GM-CSF, IFN-γ, and KC (HFD *vs* HFD-FR) are not a paradoxical result, but reflects the complexity of the beneficial effects of FR (4,10).

Third, there were changes in the blood levels of cytokines in both HFD *vs* HFD-FR and HCD *vs* HCD-FR. This change was observed only for IL-12. Our results are in agreement with those of de Oliveira et al. (15), who recently demonstrated reduced IL-12 levels in peritoneal macrophages in mice submitted to food restriction.

IL-12 is an important pro-inflammatory cytokine produced by antigen-presenting T cells, such as dendritic cells, macrophages, and natural killer cells, which plays a critical role in cell-mediated immunity. It has been observed that plasma concentrations of IL-12 are elevated in diabetes, and may contribute to atherosclerotic plaque formation and the development of macrovascular complications (16). Current data also suggest that IL-12 plays a critical role in the pathogenesis of type 2 diabetes (17) and cardiovascular disease (18). In addition, IL-12 administration accelerates the onset of autoimmune insulinitis and diabetes via increased activity of TH1 cells in non-obese diabetic mice (19).

IL-12 has emerged as an important pathway for chronic inflammation with very important clinical implications. For example, ustekinumab, a monoclonal antibody that blocks the p40 subunit of IL-12 and prevents the interaction of IL-12 with its receptor, is currently approved in the management of psoriasis, arthritis, and Crohn's disease (20).

In conclusion, only serum IL-12 decreased after 56 days of food restriction in both HFD and HCD groups, suggesting that IL-12 could represent a suitable biomarker for the beneficial effect of food restriction not only in mice receiving a high fat diet but also for mice receiving a high carbohydrate diet.
